# Association of lower urinary tract symptoms and geriatric nutritional risk index in men: a cross-sectional study based on NHANES

**DOI:** 10.3389/fmed.2024.1356921

**Published:** 2024-06-21

**Authors:** Tianyun Zheng, Huaibin Sun, Yueqing Tang, Yuan Zeng, Lei Yan

**Affiliations:** ^1^Department of Urology, Qilu Hospital of Shandong University, Jinan, China; ^2^Department of Organ Transplantation, Qilu Hospital of Shandong University, Jinan, China

**Keywords:** lower urinary tract symptoms, geriatric nutritional risk index, nocturia, nutrition, frailty

## Abstract

**Background:**

Despite previous literature exploring the factors influencing lower urinary tract symptoms (LUTS), few studies have examined the relationship between nutritional status and LUTS.

**Objectives:**

The objective of this research was to evaluate the relationship between LUTS and Geriatric Nutritional Risk Index (GNRI) in middle-aged and older men.

**Methods:**

We included 2,607 men in the NHANES 2005–2006 and 2007–2008 cycles for cross-sectional analysis. We screened for LUTS based on four specific questions on the relevant questionnaire. We calculated GNRI according to the relevant calculation formula and included other covariates. Multivariate logistic analysis using GNRI as the principal independent variable and adjusting for other covariates were used to determine the association with LUTS, nocturia, and daytime LUTS.

**Results:**

According to the responses to the questionnaire, out of 2,607 eligible participants, 471 had LUTS, 906 had nocturia, and 819 had daytime LUTS. In the unadjusted regression model, LUTS (OR = 0.93, 95% CI = 0.91–0.96, *p* < 0.001), nocturia (OR = 0.90, 95% CI = 0.88–0.93, *p* < 0.001), and daytime LUTS (OR = 0.96, 95% CI = 0.94–0.99, *p* = 0.002) were significantly negatively associated with GNRI. After adjustment by adding covariates, LUTS (OR = 0.97,95% CI =0.94–0.99, *p* = 0.026) and nocturia (OR = 0.94, 95% CI =0.91–0.93, *p* < 0.001) were significantly negatively associated with GNRI.

**Conclusion:**

Low GNRI was associated with the development of LUTS. In the prevention and treatment of LUTS, urologists should consider the impact of nutritional status on LUTS, and interventions for nutritional status may prevent and improve LUTS.

## Introduction

Lower urinary tract symptoms (LUTS), as a common series of symptoms in men, are statistically 40 percent prevalent in men over 50, and even more than 60 percent prevalent in men over 60 (1). LUTS can seriously affect men’s physical and mental health (2, 3). Many studies have shown that LUTS are no less of a threat to men’s health than diabetes, cardiovascular disease, and other common chronic diseases (4, 5). In addition, previous research indicated a close association between LUTS and depression (6). LUTS not only affect patients’ daily life, but also places a heavy burden on health insurance systems throughout the country. In the United States, billions of dollars are spent annually on the treatment of LUTS (7, 8). Therefore, effective prevention and reasonable management of LUTS is a serious challenge for urologists.

Currently, it is widely accepted that LUTS are a series of symptoms caused by functional disorders such as benign prostatic hyperplasia (BPH), including dysuria, urgency, and frequency of urination (3). Although mechanical obstruction caused by BPH is the main cause of LUTS, many studies have also shown that obesity, diabetes, sleep disorders, and poor bowel habits also worsen LUTS (9–12). However, there is little literature discussing the relationship between LUTS and nutritional status.

Malnutrition is a principal problem for many middle-aged and older men, and poor nutritional status is strongly associated with numerous diseases (13, 14). In recent years, to evaluate nutritional status, many nutritional indicators have been invented to estimate. The Geriatric Nutritional Risk Index (GNRI) has become one of the mainstream indicators for identifying malnutrition in middle-aged and older people due to its simple calculation and easy availability of the required indicators (15). Numerous research confirmed that the GNRI is not only a good assessment of nutritional status, but also displays good performance in disease diagnosis and prognosis assessment, providing a reliable reference for urinary system disease diagnosis and treatment (16, 17). Recently, frailty has begun to be explored in various diseases. Frailty is often accompanied by malnutrition, which, in turn, can lead to symptoms of frailty such as muscle atrophy and decreased bone density (18). Bauer et al. noted that frailty exacerbates LUTS in a multicenter study (19). This suggests that nutritional status may have some effect on LUTS. Therefore, our research seeks to explore the potential association between LUTS and GRNI, providing nutritional insights for the prevention and management of LUTS.

## Materials and methods

### Study population origin and screening

The National Health and Nutrition Examination Survey (NHANES) is a comprehensive representative survey that collects multidimensional health information on children and adults. We included data from two cycles (2005–2006 and 2007–2008) because only these two cycles had detailed questionnaire data to assess LUTS. Since the age of the relevant questionnaire was defined as men over 40 years old, men over 40 years old who received the questionnaire were the group of our study. The detailed exclusion requirements were as follows: (1) Participants who did not answer all the questions in the questionnaire or refused to answer the questionnaire; (2) Participants whose height, weight, or albumin were not measured; (3) Participants with incomplete covariate data.

### Evaluation of LUTS

The assessment of LUTS in this study was based on two questionnaires: “Kidney Conditions—Urology” and “Prostate Conditions.” We assessed symptoms based on the following four questions: (1) urinary hesitancy, “Have trouble starting to urinate?” (2) difficult to empty urine completely, “After urinating does bladder feel empty?” (3) nocturia, “How many times urinate in night.” (4) urinary frequency, “How often have urinary leakage.” Daytime LUTS was defined as participants answering yes to the first or second question, or leaking urine more than or equal to one. Nocturia was defined as participants urinating twice or more during the night. The full definition of LUTS is that a participant has two or more of the above four symptoms.

### Definition of GNRI

According to the Lorentz formula, the ideal weight for men is calculated as follows: height (cm) – 100 – [(height – 150)/4]. The detailed GNRI calculation formula is as follows: GNRI = [41.7 × present weight (Kg)/ideal weight (Kg)] + [1.489 × serum albumin (g/L)]. When [present weight (Kg)/ideal weight (Kg)] ≥ 1, the ratio is considered equal to 1.

### Collection and definition of other data

The relevant baseline information collected in this study is as follows: age, ethnicity, poverty income ratio (PIR), insurance, drinking status, smoking status, educational level, sleep disorder, comorbidity index, and diabetes. The comorbidity index is the sum of common chronic diseases, which includes cancer, congestive heart failure, hypertension, emphysema, congestive heart failure, and chronic bronchitis. We analyzed diabetes separately and did not include it in the comorbidity index. The comorbidity index was divided into four grades including 0, 1, 2, and more than 3. Smoking status was categorized as current, former, and never. Drinking status was categorized as current, former, and never. PIR less than 1 was considered poor and greater than or equal to 1 was considered non-poor. Smoking status was the categorical variable, and the full definition of smoking status was as follows: those who had smoked less than 100 cigarettes in their lifetime were defined as never, those who had smoked more than 100 cigarettes in their lifetime but were not current smokers were defined as former, and those who were still current smokers were defined as current. Drinking status was the categorical variable, and the full definition of drinking status was as follows: those who had fewer than 12 drinks in their lifetime were defined as never, those who had more than 12 drinks in their lifetime but had not consumed alcohol in the past 12 months were defined as former, and those who had consumed alcohol in the past 12 months were defined as current.

### Statistical analysis strategy

In order to make the sample of the database nationally representative, NHANES uses a complex four-level probability sampling, including: counties, segments, households, and individuals. Specific subgroups were oversampled to ensure that sufficient population subgroups were included in the study and that estimates of health status indicators for specific subgroups were reliable and accurate. The NHANES uses sample weights and complex sampling methods to make the data of the participants more representative. Therefore, this study selects appropriate sample weights and uses an R package named “survey” to deal with complex sample design and weighted data. Quantitative data with normal distribution were displayed as mean (SD) and analyzed by t-test. The skew distribution data were represented by the median (IQR) and analyzed by the Mann–Whitney test. The classified data were represented by a percentage and analyzed by the Chi-square test. Unadjusted logistic regression analysis was performed first, and then adjusted logistic regression analysis with relevant covariates was performed to determine the relationship between the main variables and the outcome variables. When *p* < 0.05 was considered statistically significant, all statistical results were bilateral.

## Results

### Distribution of baseline data

**Figure 1:**
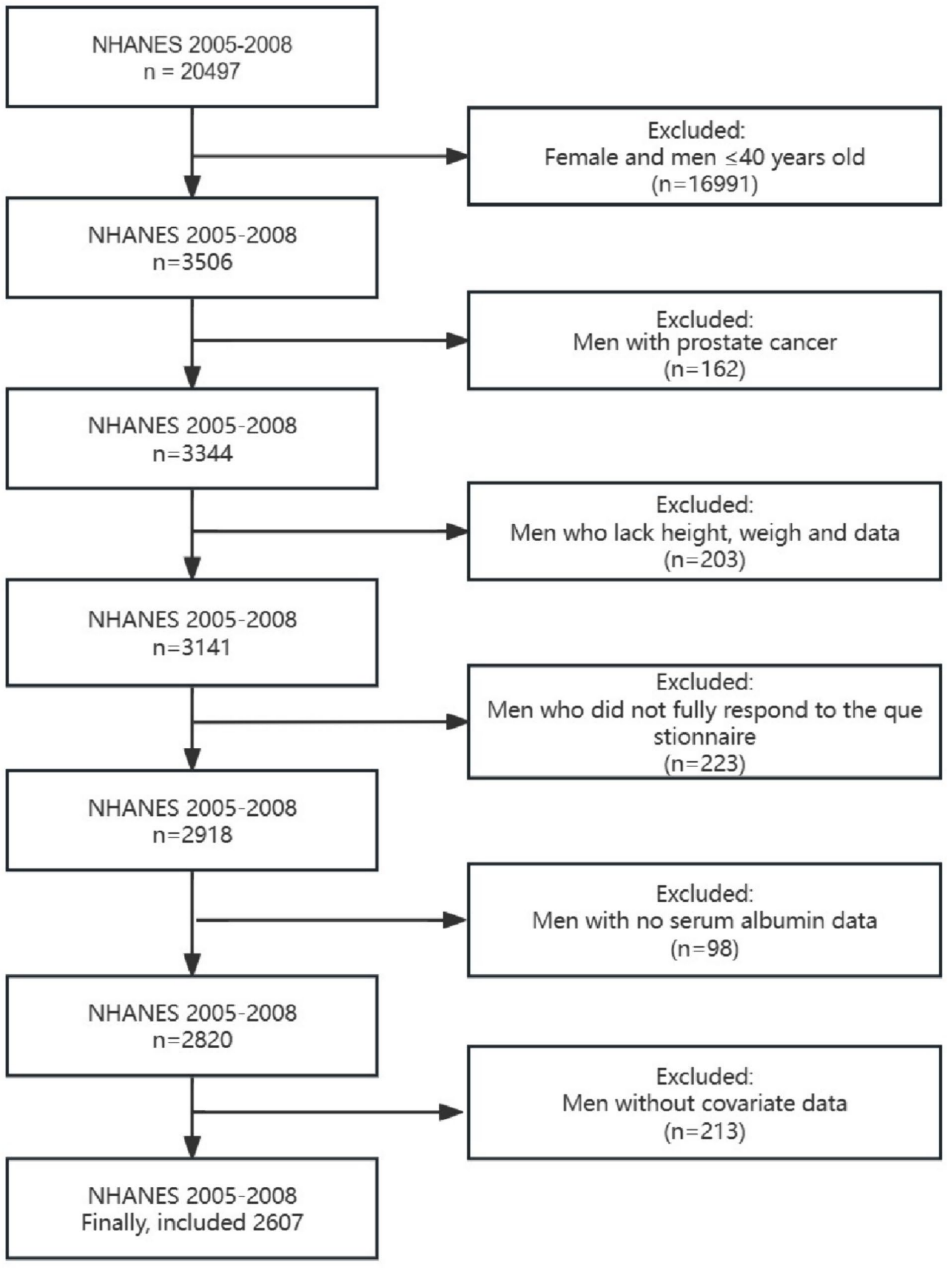
Flow chart for screening the final study population.

Of the 2,607 participants included who met the screening requirements (Figure 1), 471 (18.07%) were found to have LUTS, 819 (31.42%) participants were found to have daytime LUTS, and 906 (34.75%) participants were found to have nocturia. GNRI (*p* < 0.001), age (*p* < 0.001), education level (*p* = 0.002), insurance status (*p* = 0.012), smoking status (*p* < 0.001), drinking status (*p* < 0.001), sleep disorders (*p* = 0.006), diabetes (*p* < 0.001), and comorbidity index (*p* < 0.001) were statistically different in both subgroups in the baseline data (Table 1).

**Table 1:** Baseline data between participants with and without LUTS.

Variables	Overall	No LUTS	LUTS	*p* value
Age (year)	59.68 (12.54)	58.44 (12.34)	65.31 (11.95)	< 0.001
Race/Ethnicity %				
Mexican American	15.69	16.10	13.80	0.089
Other Hispanic	6.48	7.02	4.03	
Non-Hispanic White	55.70	55.01	58.81	
Non-Hispanic Black	18.99	18.73	20.17	
Other Race	3.14	3.14	3.19	
Education Level (adults) %				0.002
< High school	30.69	29.21	37.37	
High school	23.97	24.49	21.65	
>High school	45.34	46.30	40.98	
PIR %				0.08
<1	15.80	15.22	18.47	
≥1	84.20	84.78	81.53	
Height (cm)	173.99 (7.59)	174.12 (7.62)	173.39 (7.42)	0.058
Weight (kg)	87.82 (19.88)	87.81 (19.74)	87.86 (20.53)	0.965
Insurance %				0.012
No	16.00	16.85	12.10	
Yes	84.00	83.15	87.90	
Smoking status %				< 0.001
Never	38.09	40.26	28.24	
Former	39.82	38.11	47.56	
Current	22.09	21.63	24.20	
Drinking status %				< 0.001
Never	7.17	6.84	8.71	
Former	27.43	25.56	35.88	
Current	65.40	67.60	55.41	
Sleep disorder %				0.006
No	90.22	90.96	86.84	
Yes	9.78	9.04	13.16	
Diabetes %				< 0.001
No	84.50	85.81	78.56	
Yes	15.50	14.19	21.44	
Comorbidity index %				< 0.001
0	46.72	50.33	30.36	
1	36.17	34.46	43.95	
2	11.70	11.09	14.44	
≥3	5.41	4.12	11.25	
GNRI	104.45 (4.79)	104.74 (4.63)	103.14 (5.29)	< 0.001

LUTS, lower urinary tract symptoms; PIR, poverty income ratio; GNRI, geriatric nutritional risk index.

### Association of GNRI with LUTS, nocturia, and daytime LUTS

GNRI was shown by unadjusted weighted logistic regression analysis to be significantly associated with LUTS (OR = 0.93, 95% CI = 0.91–0.96, *p* < 0.001), nocturia (OR = 0.90, 95% CI = 0.88–0.93, *p* < 0.001) and daytime LUTS (OR = 0.96, 95% CI = 0.94–0.99, *p* = 0.002). Weighted multivariate logistic analyses adjusted to control for the covariates of age, race, poverty index, education, insurance status, co-morbidity index, diabetes, drinking status, and smoking status found that the GNRI was significantly associated with LUTS (OR = 0.97,95% CI =0.94–0.99, *p* = 0.026) and nocturia (OR = 0.94, 95% CI =0.91–0.93, *p* < 0.001), and not significantly associated with daytime LUTS (*p* = 0.5) (Table 2). Among the covariates age, smoking status and comorbidity index were correlated with LUTS. And age, PIR, comorbidity index, smoking status and diabetes were correlated with nocturia (Table 3).

**Table 2:** Association of GNRI with LUTS, nocturia, and daytime LUTS.

	Unadjusted model OR (95% CI)	*p* value	Adjusted model OR (95% CI)	*p* value
*LUTS*				
No	Reference		Reference	
Yes	0.93 (0.91–0.96)	< 0.001	0.97 (0.94–0.99)	0.026
*Nocturia*				
No	Reference		Reference	
Yes	0.90 (0.88–0.93)	< 0.001	0.94 (0.91–0.97)	< 0.001
*Daytime LUTS*				
No	Reference		Reference	
Yes	0.96 (0.94–0.99)	0.002	0.99 (0.96–1.02)	0.5

LUTS, lower urinary tract symptoms; OR, odd ratio. Adjusted for age, race, education level, PIR, insurance, smoking status, drinking status, sleep disorder, diabetes, comorbidity index.

**Table 3:** The influence of covariates on LUTS.

	OR (95% CI)	*p* value
*LUTS*		
Age	1.04 (1.03–1.06)	< 0.001
Comorbidity index		< 0.001
0	Reference	
1	1.70 (1.22–2.36)	
2	1.25 (1.20–2.29)	
≥3	2.83 (1.50–5.34)	
Smoking status		0.047
Never	Reference	
Former	1.52 (1.01–2.30)	
Current	1.56 (1.02–2.38)	
*Nocturia*		
Age	1.04 (1.03–1.06)	< 0.001
PIR		0.001
<1	Reference	
≥1	1.72 (1.20–2.48)	
Comorbidity index		
0	Reference	0.012
1	1.69 (1.18–2.44)	
2	1.40 (1.12–2.11)	
≥3	1.75 (1.24–3.16)	
Smoking status		0.002
Never	Reference	
Former	1.35 (1.04–1.75)	
Current	1.53 (1.16–2.02)	
Diabetes		0.03
No	Reference	
Yes	1.53 (1.012–2.35)	

LUTS, lower urinary tract symptoms; PIR, poverty income ratio; OR, odd ratio.

## Discussion

In the past few years, mechanical obstruction caused by BPH was the principal mechanism causing LUTS. Therefore, the treatment of LUTS by urologists in the past was mainly based on surgical treatment of BPH. However, with the increasing understanding of the etiology of LUTS among urologists, the etiology of LUTS is very complex, and BPH is not the only culprit of LUTS. Although there are many drugs to treat LUTS, the drug treatment process is long and requires good compliance, so many patients often give up halfway (20). Therefore, considering the distress and burden of LUTS for patients, the prevention of LUTS has gradually become the focus of urologists. Qin et al. explored the relationship between muscle mass and LUTS, demonstrating that frailty was positively correlated with LUTS (21). Many studies indicated that poor nutrition was closely related to frailty (22, 23). Malnutrition is recognized as a significant pathophysiological risk factor for frailty. Although there are many related comprehensive indicators or questionnaires, many older people cannot cooperate with the examination due to cognitive dysfunction and other reasons, which affect the credibility of the assessment. Our index requires fewer indicators, and these indicators are relatively easy to obtain and objective. Therefore, our research intended to seek the relationship between LUTS and GNRI, providing nutritional recommendations for the prevention and management of LUTS. GNRI is inversely associated with LUTS and is a protective factor, as shown in the present study, which proves that the probability of LUTS occurrence is lower under good nutritional status.

Before the advent of GNRI, the Nutritional Risk Index (NRI) was one of the indicators used to assess nutritional status. However, one study based on NHANES indicated that instrumental measurements in people older than 70 years were often inconsistent with self-reported weight (24). Therefore, the results of such calculations tend to be less reliable. GNRI avoids this error because it calculates ideal body weight in a way that makes the data more accurate. GRNI is widely adopted in the prevention and prognosis of cancer and other diseases due to its good evaluation ability (25–27). One study showed that GNRI in elderly patients was a simple and intuitive way to evaluate muscle function, especially grip strength (28). Takashi et al. analyzed data from diabetic patients and found that sarcopenia was significantly associated with low levels of GNRI (29). A previous study compared GNRI with other simple nutritional assessment indicators and found that GNRI had excellent evaluation ability compared with other indicators, especially for muscle mass and subcutaneous fat (30). Grip strength is an intuitive indicator of muscle strength and even nutritional status. One study noted a positive association between GNRI and grip strength in men (31). These studies show that the GNRI is an effective indicator of evaluating muscle mass and that a decline in muscle mass is one of the classic symptoms of frailty. Malnutrition is the initiating factor of muscle mass loss. Therefore, muscle mass appears to link nutritional status to frailty.

In a study analyzing the condition of LUTS in patients with systemic sclerosis, researchers found that muscle atrophy can lead to symptoms of overactive bladder (32). A large study showed significantly higher frailty scores among participants with nocturia than those without nocturia (33). Another study followed 247 patients with LUTS for five years and found that patients with higher frailty scores had worse LUTS symptoms over time. This study also demonstrates that LUTS, in turn, can exacerbate frailty (34). A community survey initiated by Japanese scholars indicates that oxidative stress indicators are closely related to nocturia (35). Oxidative stress activates inflammatory pathways and leads to increased degradation and decreased synthesis of proteins, which is the underlying mechanism of sarcopenia (36). In terms of inflammation and GNRI, as part of this index, albumin is associated with inflammation, so inflammation is also one of the causes (37). Besides, a study based on rural populations showed that older, frail people are vulnerable to suffering from urinary incontinence (38). Bauer et al. analyzed patients with LUTS in this center and pointed out that frailty should be considered in the initial diagnosis of LUTS (39).

The potential mechanism of malnutrition leading to LUTS may be frailty due to malnutrition. Malnutrition in middle-aged and elderly people is a major social challenge affecting population health. The imbalance between insufficient nutrient intake and energy expenditure leads to a negative nitrogen balance, which contribute to muscle atrophy followed by frail symptoms such as decreased physical function. And these are the classic signs of frailty. In addition, frail patients are often due to low muscle content and low metabolic levels, resulting in problems such as loss of appetite, exacerbating the problem of malnutrition. As a result, a vicious circle is created between poor nutrition and frailty. In addition, albumin is considered a reference index of nutritional level and inflammation (40). The activation of inflammatory pathways caused by oxidative stress will produce a large number of tumor necrosis factor and interleukin molecules, and these inflammatory factors will lead to the decrease of albumin levels (36, 41). This coincides with the relationship between LUTS and oxidative stress mentioned above. Increased vascular permeability caused by oxidative stress can also cause albumin to enter the extravascular tissue space. Therefore, oxidative stress and nutritional status are inextricably linked. European scholars have analyzed the etiology of sarcopenia and found that nutritional status plays a vital role in it. Bartali et al. (42) assessed the relationship between various indicators of frailty and malnutrition and showed that malnutrition directly leads to frailty. Elderly people with poor nutrition conditions tend to have weakened immune defenses and decreased bone mineral density, and studies have shown that such patients have significantly increased mortality (43, 44). Therefore, malnutrition directly and indirectly affects LUTS due to frailty and loss of muscle mass.

Among the covariates, age, comorbidities index, and smoking status had some influence on LUTS. As people get older, they become more likely to suffer from physical and mental illnesses, so frailty becomes more common, especially in nursing homes and hospitals. Therefore, for most diseases, age is a common factor that induces or aggravates the condition. Frail older adults tend to have multiple chronic illnesses. The large prospective study by Hanlon et al. not only affirmed the fact that there were more comorbidities in frail patients, but they also suggested that middle-aged adults with multiple chronic conditions tended to have higher mortality due to frailty (45). It proves that chronic diseases exacerbate frailty, which may also be the reason why chronic diseases affect LUTS. A large questionnaire survey from Japan showed a significantly increased risk of LUTS in men who were former or current smokers compared with men who had never smoked (46).

Combined with other research on LUTS, we realized that few research explored the relationship between LUTS and GNRI. The NHANES is a rigorous multistage cluster sampling survey design with weights assigned to each participant, so the data are well representative. In addition, the staff were professionally trained and examined the collected data, which made our study data more authentic and reliable. Our study also included covariates factors reported in other literature to influence LUTS, which made our research more comprehensive. However, our research still has some relevant limitations. First of all, due to the limitations of cross-sectional studies, the causality between GNRI and LUTS cannot be determined, and more in-depth research is needed to explore the specific mechanism. Secondly, although the questionnaire process is very rigorous, it is based on the subjective description of patients, so there is a certain degree of recall bias. Finally, although the sample capacity we included was relatively adequate, a larger study with more covariates is needed for further refinement.

## Conclusion

Our study indicated an association between GNRI and an increased risk of LUTS and nocturia. For LUTS, urologists should consider the impact of frailty due to malnutrition on LUTS. Therefore, malnourished men should be alert to LUTS, and the management of male LUTS patients should be aware of the importance of improving nutritional status. This study provided a simple and effective nutritional index for urologists to prevent and manage LUTS and made more people aware of the impact of nutritional status on LUTS. In the future, the effectiveness of nutritional intervention in the prevention and alleviation of LUTS needs further research to uncover.

## Data availability statement

Publicly available datasets were analyzed in this study. This data can be found at: https://www.cdc.gov/nchs/nhanes/index.htm.

## Ethics statement

The studies involving humans were approved by the institutional review board of the National Center for Health Statistics. The studies were conducted in accordance with the local legislation and institutional requirements. The participants provided their written informed consent to participate in this study.

## Author contributions

TZ: Conceptualization, Data curation, Formal analysis, Methodology, Writing – original draft. HS: Formal analysis, Methodology, Writing – review & editing. YT: Conceptualization, Writing – original draft. YZ: Software, Writing – original draft. LY: Supervision, Writing – review & editing.
